# The Antimicrobial Activity of a Carbon Monoxide Releasing Molecule (EBOR-CORM-1) Is Shaped by Intraspecific Variation within *Pseudomonas aeruginosa* Populations

**DOI:** 10.3389/fmicb.2018.00195

**Published:** 2018-02-08

**Authors:** Lindsey Flanagan, Rachel R. Steen, Karinna Saxby, Mirre Klatter, Benjamin J. Aucott, Craig Winstanley, Ian J. S. Fairlamb, Jason M. Lynam, Alison Parkin, Ville-Petri Friman

**Affiliations:** ^1^Department of Biology, University of York, York, United Kingdom; ^2^Department of Chemistry, University of York, York, United Kingdom; ^3^Department of Clinical Infection, Microbiology and Immunology, Institute of Infection and Global Health, University of Liverpool, Liverpool, United Kingdom

**Keywords:** biofilms, carbon monoxide releasing molecules, CORM, cystic fibrosis, polymicrobial infections, *Pseudomonas aeruginosa*, synthetic chemistry, virulence

## Abstract

Carbon monoxide releasing molecules (CORMs) have been suggested as a new synthetic class of antimicrobials to treat bacterial infections. Here we utilized a novel EBOR-CORM-1 ([NEt_4_][MnBr_2_(CO)_4_]) capable of water-triggered CO-release, and tested its efficacy against a collection of clinical *Pseudomonas aeruginosa* strains that differ in infection-related virulence traits. We found that while EBOR-CORM-1 was effective in clearing planktonic and biofilm cells of *P. aeruginosa* strain PAO1 in a concentration dependent manner, this effect was less clear and varied considerably between different *P. aeruginosa* cystic fibrosis (CF) lung isolates. While a reduction in cell growth was observed after 8 h of CORM application, either no effect or even a slight increase in cell densities and the amount of biofilm was observed after 24 h. This variation could be partly explained by differences in bacterial virulence traits: while CF isolates showed attenuated *in vivo* virulence and growth compared to strain PAO1, they formed much more biofilm, which could have potentially protected them from the CORM. Even though no clear therapeutic benefits against a subset of isolates was observed in an *in vivo* wax moth acute infection model, EBOR-CORM-1 was more efficient at reducing the growth of CF isolate co-culture populations harboring intraspecific variation, in comparison with efficacy against more uniform single isolate culture populations. Together these results suggest that CORMs could be effective at controlling genetically diverse *P. aeruginosa* populations typical for natural chronic CF infections and that the potential benefits of some antibiotics might not be observed if tested only against clonal bacterial populations.

## Introduction

The rapid emergence of multidrug-resistant bacteria is a global problem that is predicted to cause 10 million deaths per year by 2050 (O'Neill, 2014). Antibiotic resistance often evolves very quickly via *de novo* mutations and horizontal gene transfer (Normark and Normark, 2002), and as a result, antibiotic discovery has not been able to replace all of the antibiotics that have now become ineffective (Brown and Wright, 2016). New methods and approaches for treating bacterial infections are thus urgently required.

In recent years, carbon monoxide has emerged as a new potential therapeutic due to its properties as a homeostatic and cytoprotective molecule with important signaling capabilities (Motterlini and Otterbein, 2010). Carbon monoxide can be delivered via carbon monoxide releasing molecules (CORMs), which are small molecules that release carbon monoxide in response to certain environmental triggers such as enzymes (Stamellou et al., 2014) or light (Jimenez et al., 2016). Nobre et al. first investigated the effect of CORMs on bacteria (Nobre et al., 2007) and found that CORM-2 and CORM-3 reduced the number of colony-forming units of *Escherichia coli* in minimal salts media and *Staphylococcus aureus* in Luria Broth (LB) media (Nobre et al., 2007). The CORM effects were stronger in near-anaerobic conditions and the activation of CORM required direct contact between the molecule and its cellular targets (Nobre et al., 2007). Moreover, the effect of CORM-3 on *Pseudomonas aeruginosa* wild type strain PAO1 was investigated by Desmard et al. (2009), who found that treatment with the CORM reduced bacterial densities and increased the survival of immunocompromised mice during an infection. It has also previously been found that CORM-2 effectively reduces the densities of *P. aeruginosa* planktonic and biofilm cultures with wild type and clinical strains (Murray et al., 2012). Another study found that manganese-based Trypto-CORM is able to inhibit the growth of *E. coli* when exposed to photochemical stimulus (Ward et al., 2014), while in the dark it is active against *Neisseria gonorrhoeae* (Ward et al., 2017). In both cases, control experiments indicate that the CO liberated from the metal is responsible for the observed behavior. However, most of the studies thus far have concentrated on exploring CORM effects on relatively short time span (<24 h). Furthermore, although it has been established that many infections are polymicrobial, and that clinical bacterial pathogens can respond differently to CORMs than laboratory strains, no studies have explored CORM effects on bacterial co-cultures.

Cystic fibrosis (CF) is a genetically inherited disease which affects 1 in 2,000 to 3,000 newborn infants in the EU (Who.Int, 2010). Patients with CF often develop a thick mucus in the lungs which they are unable to clear (Flume et al., 2010). This mucus makes patients susceptible to frequent and recurring bacterial chest infections and the presence of *P. aeruginosa* is often associated with increasing morbidity and loss of lung function (Pritt et al., 2007). One of the key features of *P. aeruginosa* is its capability to rapidly adapt to the lung environment and to become highly resistant to the antibiotics that are used to treat infections (Smith et al., 2006; Poole, 2011; Folkesson et al., 2012; Winstanley et al., 2016). As a result, *P. aeruginosa* populations show high levels of genetic variation within and between CF patients (Marvig et al., 2013; Williams et al., 2015; O'Brien et al., 2017). This includes phenotypic and genomic heterogeneity within genetically-related populations of *P. aeruginosa* derived from the same clonal lineage (Mowat et al., 2011; Workentine et al., 2013; Williams et al., 2015). This variation might also affect the applicability of potential alternative therapies if it is linked with bacterial life-history traits that relate to potential resistance mechanisms.

Here we synthesized and characterized a water-soluble CORM (EBOR-CORM-1), [NEt_4_][MnBr_2_(CO)_4_], and tested its effectiveness against *P. aeruginosa* strain PAO1 and a selection of *P. aeruginosa* CF isolates originating from a single sputum sample from the lungs of a CF patient, namely patient CF03 from previously published studies (Mowat et al., 2011; Williams et al., 2015). Based on genome sequence data presented in a previous study, these CF isolates were classified into two genetically distinct Liverpool Epidemic Strain (LES) lineages, A and B (Williams et al., 2015, 2016), that differ regarding their virulence traits (O'Brien et al., 2017). These genetically diverged lineages have been shown to commonly coexist within individual patients and to share mutations via homologous recombination that potentially help strains to adapt to the airway during chronic infection (Williams et al., 2015). However, the implications of within-patient genetic variation have been seldom considered in the context of antimicrobial therapies. We hypothesized that effects of EBOR-CORM-1 could vary between different clinical isolates and lineages, and that the susceptibility of isolates could be linked to expression of some other bacterial virulence factors. We found that the CORM was effective in reducing both planktonic and biofilm cells of strain PAO1 in a density-dependent manner. However, CORM effects were more varied and generally weaker against clinical CF isolates. Regardless, CORM efficiently reduced the growth of CF strain lineage co-cultures, which suggest that CORMs could be effective at controlling genetically diverse *P. aeruginosa* infections.

## Materials and methods

### Synthesis and properties of [NEt_4_][MnBr_2_(CO)_4_], EBOR-CORM-1

EBOR-CORM-1 was synthesized as described previously (Angelici, 1964): Mn(CO)_5_Br (466 mg, 1.69 mmol) and 330 mg (1.57 mmol) of [(C_2_H_6_)_4_N]Br were heated in 18 mL of absolute methanol under a nitrogen atmosphere at 50°C for 1 h. The methanol was then evaporated from the orange solution at the above temperature. The remaining yellow solid was dissolved in 40 mL of chloroform, and the solution was filtered under nitrogen. After adding 200 mL of hexane to the filtrate, the cloudy solution was allowed to stand under nitrogen for 2 h. The air-stable yellow crystals were separated by filtration, washed with hexane, and dried under vacuum giving a yield of 88% (636 mg). The compound was characterized *via* solid state IR spectroscopy recorded using a KBr disk. Four main bands were seen at 2,090, 2,001, 1,984, and 1,942 cm^−1^ and a small shoulder was seen at 1,897 cm^−1^. This is consistent with the literature values (Angelici, 1964). In a chloroform solution of CORM four distinct bands were observed at 2,092, 2,015, 1,987, and 1,943 cm^−1^, again this is similar to previously reported literature values (Angelici, 1964). The change in the number of carbonyl bands between the solid and solution phase measurements typically reflects that different orientations are present in the solid state. The stability of the CORM in the solid state was tested by heating a sample to 50°C and running ATR IR spectra at 1 h intervals.

Infrared detection of CO release from EBOR-CORM-1 following dissolution in different solvents was conducted by dissolving 12 mg of CORM in 4 mL of solvent in a 25 mL round-bottomed flask attached to a vacuum evacuated gas IR cell via a closed tap. After 1 h of stirring the flask, the tap was opened to enable gas from the headspace of the flask to enter the IR cell. Carbon monoxide could then be identified via the distinctive gaseous IR signature of a double band, with fine rotational splitting, centered at 2,150 cm^−1^ (Klein et al., 2014). The impact of different solvents can be quantified by comparison of the intensity of the CO bands to those from CO_2_, which is assumed to act as an effective internal standard.

The release of CO from EBOR-CORM-1 following dissolution in water was also followed via solution phase monitoring of the metal complex's IR bands. In contrast to chloroform, when EBOR-CORM-1 was first dissolved in water only two main IR bands were observed at 2,050 and 1,943 cm^−1^. In order to investigate activity of EBOR-CORM-1 in liquid culture media, we compared the effects of active and “inactivated” CORM on the growth of PAO1 strain in LB media as described previously (Murray et al., 2012). Briefly, CORM was inactivated by storing a 2 mM CORM stock LB solution (10% v/v of standard LB concentration, i.e., the same that was used in all the experiments; see below) at room temperature for 24 h. To estimate the effect of CORM inactivation on PAO1 growth, we added 50 μL of freshly prepared 2 mM CORM, 50 μL of inactivated 2 mM CORM or 50 μL 10% v/v LB (control) to 150 μL of PAO1 starter culture on 96-well microplate. All treatments were replicated five times and PAO1 growth monitored for 8 h at 37°C with a spectrophotometer (OD 600 nm; Tecan Infinite).

### Bacterial strains and culture media

In this study we used *P. aeruginosa* strain PAO1 (ATCC 15692), the earliest archived isolate of the Liverpool Epidemic strain, LESB58 (Winstanley et al., 2009), and 19 clinical *P. aeruginosa* LES isolates from the same sputum sample of a chronically infected CF patient (Williams et al., 2015). The CF lung LES isolates originate from the sputum sample of one patient, identified as patient CF03 in previous studies, and consist of two genetically separate lineages A and B (Williams et al., 2015). Lineage A was represented by six isolates, namely isolates: 2, 5, 10, 19, 23, and 25. Lineage B was represented by 13 isolates, namely isolates: 1, 6, 8, 17, 24, 26, 28, 32, 33, 34, 35, 36, and 37. Clinical isolates were collected with the consent of the patient and under institutional human investigation approval. All strains and isolates of *P. aeruginosa* were routinely cultured in liquid or solid LB media containing 10.0 g tryptone, 5.0 g yeast extract and 10.0 g NaCl in 1 L of ultra-pure water (final pH adjusted to 7.0 and 15 g of agar was used for solid media). For all experiments, starter cultures were prepared from cryofrozen stocks by streaking frozen stock culture onto LB plates. After 24 h growth, a single colony was selected and inoculated into 5 mL of liquid LB and grown overnight in a shaking incubator at 37°C in 50 mL centrifuge tubes. Overnight cultures were centrifuged at 4,000 rpm (11.5 *g*) for 15 min (Eppendorf), the resultant pellets were suspended in 10% LB and bacterial densities adjusted to optical density at 600 nm of 0.066 before use (OD 600 nm), equalling approximately 1 × 10^8^ cells mL^−1^.

### Measuring the effects of EBOR-CORM-1 concentration on *P. aeruginosa* PAO1 strain

We measured the effect of CORM concentration on *P. aeruginosa* PAO1 in four different ways. First, we examined how EBOR-CORM-1 affects PAO1 growth after both 8 and 24 h of inoculation in 10% LB media (bacteria and CORM inoculated at the same time). Additionally, we measured how effective EBOR-CORM-1 is at clearing both established planktonic and biofilm PAO1 cultures (bacteria pre-grown before adding EBOR-CORM-1). All measurements were conducted on 96-well microplates and each treatment was replicated 5 times. A variety of EBOR-CORM-1 concentrations were tested by first preparing a 4 mM CORM stock solution (dissolving EBOR-CORM-1 in 10% LB media by vortexing for 30 s and sonicating for 1.5 min). The stock solution was then sterilized with syringe filtration and serially diluted to result in 1, 0.5, 0.25, 0.125, and 0 mM (control) EBOR-CORM-1 concentrations and 1 × 10^8^ PAO1 cells mL^−1^ with final volume of 200 μL of media. The microplate was then incubated at 37°C for 24 h.

All replicate populations were sampled at 8 and 24 h after the start of the experiment (20 μL of samples) and serially diluted in sterile PBS on microplates to quantify the number of living vs. dead cells by flow cytometry. Briefly, DAPI (4′,6-diamidino-2-phenylindole for dead and living cells) and PI (Propidium iodide for dead cells) fluorescent stains (both from Sigma-Aldrich) were added to microplate wells with diluted bacterial samples at concentrations of 1 μg/mL and 50 μM, respectively. Plates were then incubated at room temperature for 1 h before measuring cell densities with a Cytoflex flow cytometer and the CytExpert program. Every well was sampled for 60 s at fast speed setting. Gating of live and dead cells was performed by monitoring DAPI staining on the PB450 channel with the 405 nm laser, and PI staining on the ECD channel of the 488 nm laser. Number of living cells was determined as total cells (DAPI)–dead cells (PI).

To quantify the effects of EBOR-CORM-1 on established planktonic and biofilm cultures, PAO1 was first grown in the absence of CORM at 37°C for 48 h. Cell cultures were then inoculated with stock CORM solution to reach the same final concentrations as above: 1, 0.5, 0.25, 0.125, and 0 mM (control) of CORM. The plate was incubated for four more hours at 37°C before sampling (20 μL), serial dilution and flow cytometry as described above. To quantify effects of EBOR-CORM-1 on biofilm, crystal violet was added to the remaining cell cultures at 10% v/v. After 15 min of incubation, the plate was rinsed with deionised water and solubilised with 228 μL ethanol per well. The biofilm was quantified by measuring absorbance at 600 nm.

### Measuring the effects of EBOR-CORM-1 on clinical *P. aeruginosa* isolates in mono- and co-cultures

Similar to the PAO1 strain experiments, we measured the effect of EBOR-CORM-1 on clinical *P. aeruginosa* isolates after 8 and 24 h of inoculation in 10% LB media. We also measured the impact of growing the isolates in the absence of EBOR-CORM-1 for 48 h and then applying EBOR-CORM-1 for 4 h using both flow cytometry and crystal violet staining. We used only one EBOR-CORM-1 concentration, 0.5 mM, which resulted in clear reduction of PAO1 cultures (see Results) alongside control treatment (no CORM).

In addition to measuring the effects of EBOR-CORM-1 in monocultures of each clinical isolate, we also quantified the effect of the CORM on mixtures of the CF clinical isolates from patient CF03. First, we prepared the clinical isolate starter cultures as described above, then we mixed the standardized monocultures together in three different ways: as a whole mix (all isolates mixed together in equal proportions), lineage A mix (all isolates classified as lineage A mixed together in equal proportions) and lineage B mix (all isolates classified as lineage B mixed together in equal proportions). All final mixes contained approximately 1 × 10^8^ cells mL^−1^ before the application of 0.5 mM of EBOR-CORM-1. Each experiment was replicated 5 times. After 24 h growth at 37°C, bacterial densities were measured by using a Tecan infinite spectrophotometer: optical density measurements correlate well with the proportion of living cells measured by flow cytometry (Supplementary Figure 1).

### Characterizing bacterial virulence and growth

To characterize production of the virulence factors pyocyanin and pyoverdine, all clinical isolates were grown in 200 μL of 10% LB media in round-bottomed 96-well microplates for 48 h at 37°C (no shaking). After incubation, we measured the bacterial densities (OD 600 nm) and centrifuged the microplate for 10 min. at 4,000 rpm (11.5 g) in a swing rotor Eppendorf centrifuge. To measure pyocyanin and pyoverdine production, 150 μL of the supernatant of each well was transferred to flat-bottomed 96-well microplates and the absorbance spectrum measured with a spectrophotometer (Tecan infinite). Per capita pyocyanin production was measured for each isolate by measuring the absorbance of supernatant at 691 nm, and then standardizing by bacterial OD (Reszka et al., 2004). Per capita production of the iron-chelating siderophore, pyoverdine, was measured by using excitation-emission assay (O'Brien et al., 2017) where the fluorescence of each supernatant well was measured at 470 nm following excitation at 380 nm, using a Tecan infinite M200 pro spectrophotometer. Also, OD was measured at 600 nm to quantify the ratio fluorescence/OD as a quantitative measure of per capita pyoverdine production (O'Brien et al., 2017). The isolate biofilm production was measured as described previously and growth as maximum density and growth rate h^−1^ during 24-h growth period. Lastly, we also measured the *in vivo* virulence of each isolate by using wax moth model as described previously (O'Brien et al., 2017).

### Testing EBOR-CORM-1 antimicrobial activity in wax moth model *in vivo*

To test the efficacy of EBOR-CORM-1 to constrain bacterial infections *in vivo*, we used a wax moth larvae model (*Galleria mellonella* [Lepidoptera: Pyralidae], Livefood UK Ltd.) and followed the infection methodology described previously (O'Brien et al., 2017). We chose three strains for infection experiments: PAO1, LESB58 and isolate 36 (Lineage B) from the clinical sample collection. Before infection, we first grew the selected *P. aeruginosa* isolates for 24 h at 37°C and subsequently diluted all cultures to approximately similar densities (equalling approximately 1 × 10^6^ cells mL^−1^ in 0.8% w:v NaCl). The virulence of every isolate was then tested in 16 independent wax moth larvae. We also infected 16 larvae with 0.8% w/v NaCl salt solution to control for the damage caused by the injection itself. The larvae were injected with either 20 μL of one bacterial solution or NaCl buffer (“non-infected”) between the abdominal segments six and seven with a 1 mL Terumo syringe. After 2 h, 8 larvae from each bacterial infection or non-infection group were treated with 20 μL injection of 500 μM EBOR-CORM-1, and the other 8 were injected with 0.8% w:v NaCl salt solution (control placebo) in the same location where the bacteria were originally injected. After infection, larvae were placed on individual wells of 24-well cell culture plates and the survival was monitored at 2-h intervals for 3 days at 37°C. Larvae were scored as dead when they did not respond to touch with forceps. Larvae that were still alive after 7 days from the infection were given a time of death of 168 h. Every bacterial isolate was tested for three times. It was concluded that the EBOR-CORM-1 injection alone did not affect larval survival in the absence of bacteria (mortality similar between non-infected CORM-injected larvae and non-infected CORM-free larvae: 5–10%).

### Statistical analysis

All data were analyzed with Generalized Mixed Models (factorial ANOVA) or regression analysis where bacterial densities (**Figures 2**, **3**, and **4B**) or trait values (**Figure 4A**; Supplementary Figure 4) were explained with the presence and/or concentration of EBOR-CORM-1, CF isolate identity (isolate number) or CF lineage (A or B). All proportional data (%) were arcsine transformed before the analysis to meet the assumptions of parametric models.

## Results

### Chemistry of EBOR-CORM-1

**Figure 1:**
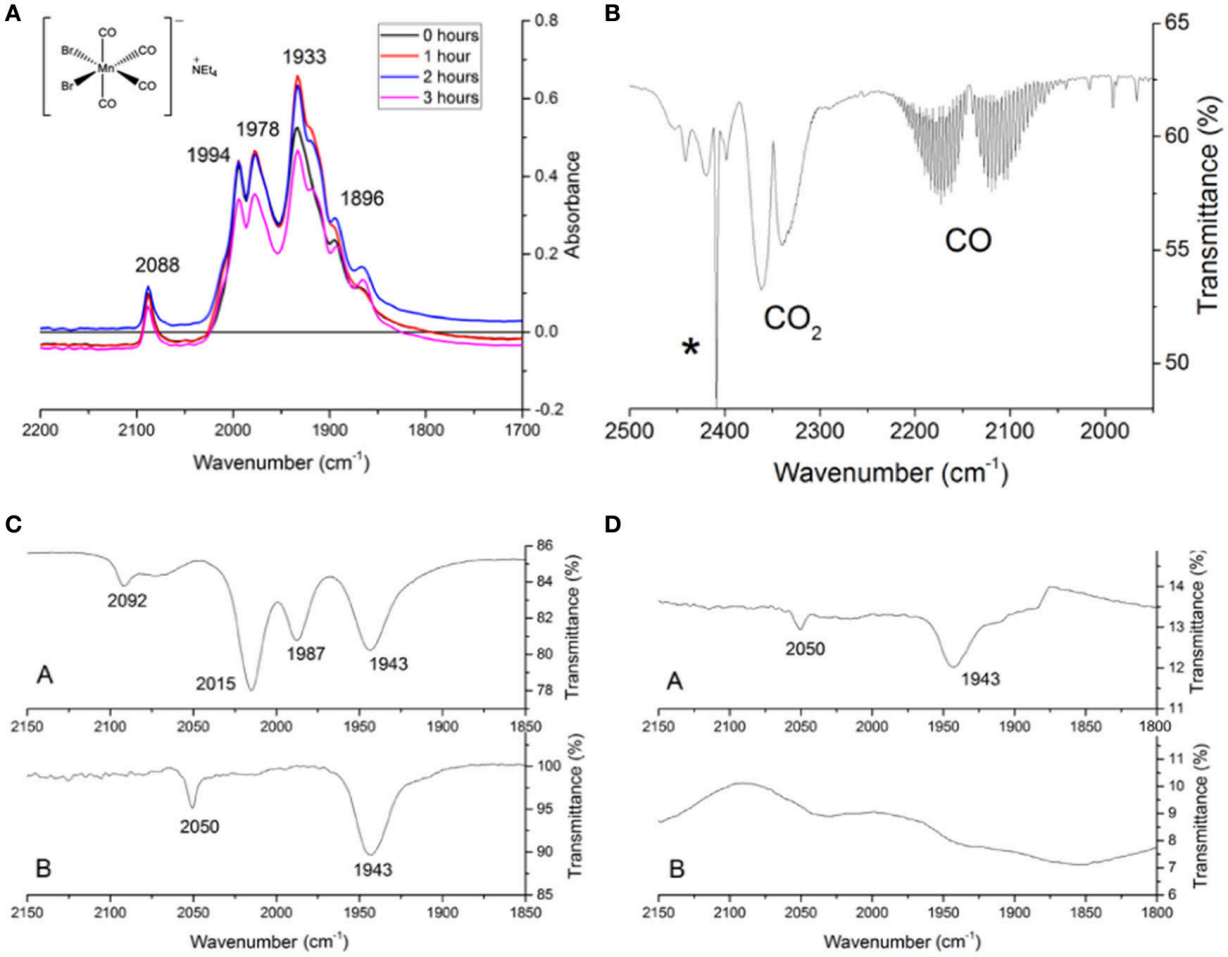
The stability of EBOR-CORM-1. **(A)** shows the IR spectra of EBOR-CORM-1 upon heating at 50°C for 0 h (black line), for 1 h (red line), for 2 h (blue), and for 3 h (pink). The structure of the EBOR-CORM-1 is shown in inset on the left. **(B)** shows the gas phase IR spectra obtained from EBOR-CORM-1 in chloroform with added water where the ^*^ indicates a band from chloroform. **(C)** shows the IR spectra of EBOR-CORM-1 in chloroform (top) and water (bottom) and **(D)** the IR spectra of CO in water after 1 min (top) and 90 min (bottom) dissolution. All wavenumbers are given in cm^−1^.

The stability of EBOR-CORM-1 in the solid state was demonstrated by heating a sample of solid to 50°C in air, and showing that there is very little difference in the carbonyl bands observed in ATR IR spectra measured at 1 h intervals over a 3-h period (Figure 1A). In contrast, gas phase infrared analysis proved that CO release from EBOR-CORM-1 can be triggered by dissolution in water, phosphate buffer or LB media, or addition of water to a solution of the compound in an organic solvent (Figure 1B).

Solution phase monitoring of the CO stretches of the compound showed that there was no reaction with water over short periods of time, since dissolving EBOR-CORM-1 in water, immediately re-drying it on a vacuum line and then re-dissolving the resultant solid in chloroform yielded an IR spectrum which matched that of the as-purified compound in chloroform (Figure 1C). The only two observed IR bands in the CORM spectrum in water (2,050 and 1,943 cm^−1^) were therefore attributed to the molecular symmetry of the hydrated complex, rather than an immediate loss of CO upon contact with water. However, after 90 min in water, a loss of these carbonyl bands was observed, and this was attributed to the release of all the CO from the complex (Figure 1D).

### EBOR-CORM-1 activity against planktonic and biofilm cells of *P. aeruginosa* PAO1

**Figure 2:**
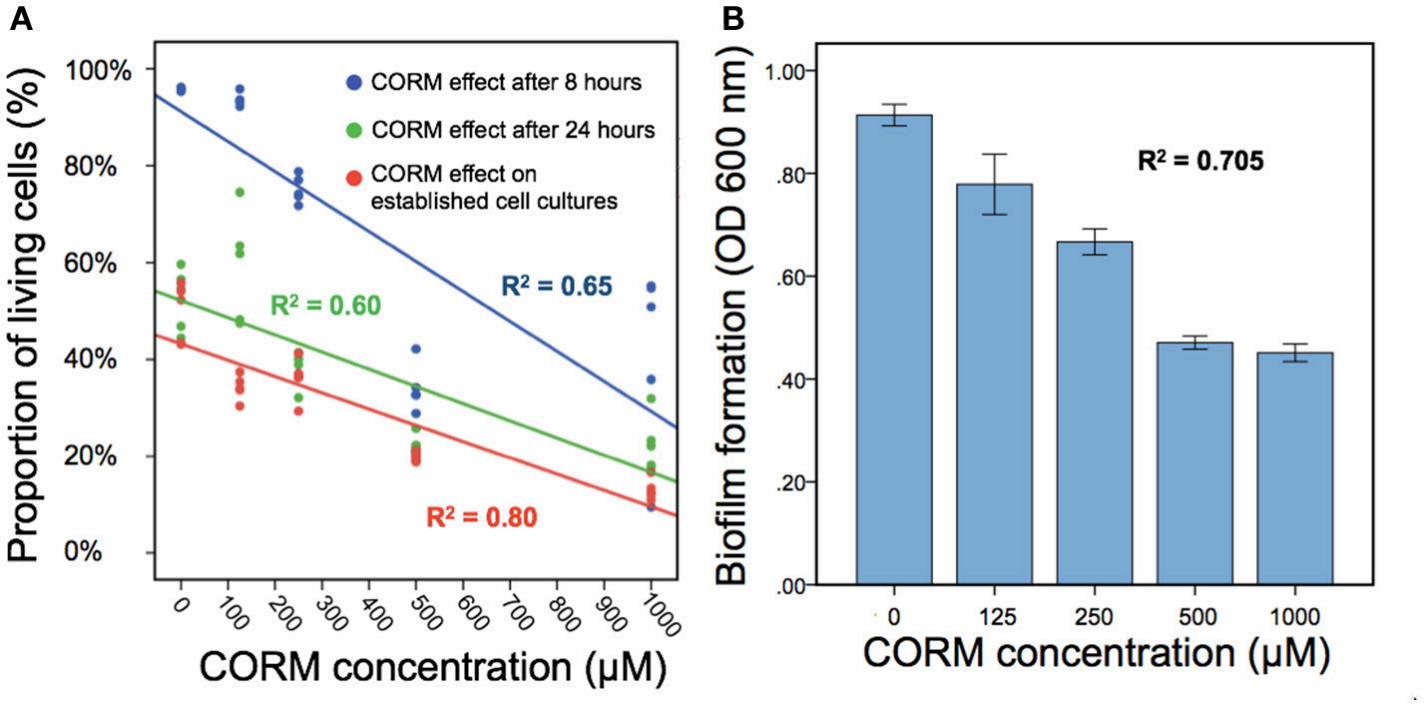
The EBOR-CORM-1 effects on planktonic and biofilm cells of *P. aeruginosa* PAO1. In **(A)**, different lines denote for cell densities after 8 h (blue line) and 24 h (green line) of EBOR-CORM-1 application and EBOR-CORM-1 effects on established cell cultures (red line) in different CORM concentrations. **(B)** shows EBOR-CORM-1 effects on PAO1 biofilms in different CORM concentrations. The R^2^ denotes for the fit of regression with our data, and in **(B)**, bars denote for ±1 s.e.m.

We found that applying EBOR-CORM-1 had generally negative effects on *P. aeruginosa* PAO1 growth both after 8 and 24 h of application [*F*_(4, 25)_ = 50.9, *p* < 0.001 and *F*_(4, 25)_ = 31.8, *p* < 0.001 for proportion of living cells after 8 and 24 h, respectively, Figure 2A] and that these negative effects increased along with the increasing concentration of applied EBOR-CORM-1 [regression analysis: *F*_(1, 24)_ = 43, *p* < 0.001 and *F*_(1, 24)_ = 35, *p* < 0.001 for proportion of living cells after 8 and 24 h, respectively, Figure 2A]. Similarly, EBOR-CORM-1 was highly effective against both established planktonic and biofilm *P. aeruginosa* PAO1 cultures [*F*_(4, 25)_ = 77.5, *p* < 0.001 and *F*_(4, 25)_ = 39.5, *p* < 0.001, respectively, Figures 2A,B] and the antimicrobial activity of the CORM increased in a density-dependent manner [regression analysis: *F*_(1, 24)_ = 92, *p* < 0.001 and *F*_(1, 24)_ = 54, *p* < 0.001, respectively, Figures 2A,B].

### EBOR-CORM-1 activity against planktonic and biofilm cells of clinical *P. aeruginosa* cystic fibrosis isolates

**Figure 3:**
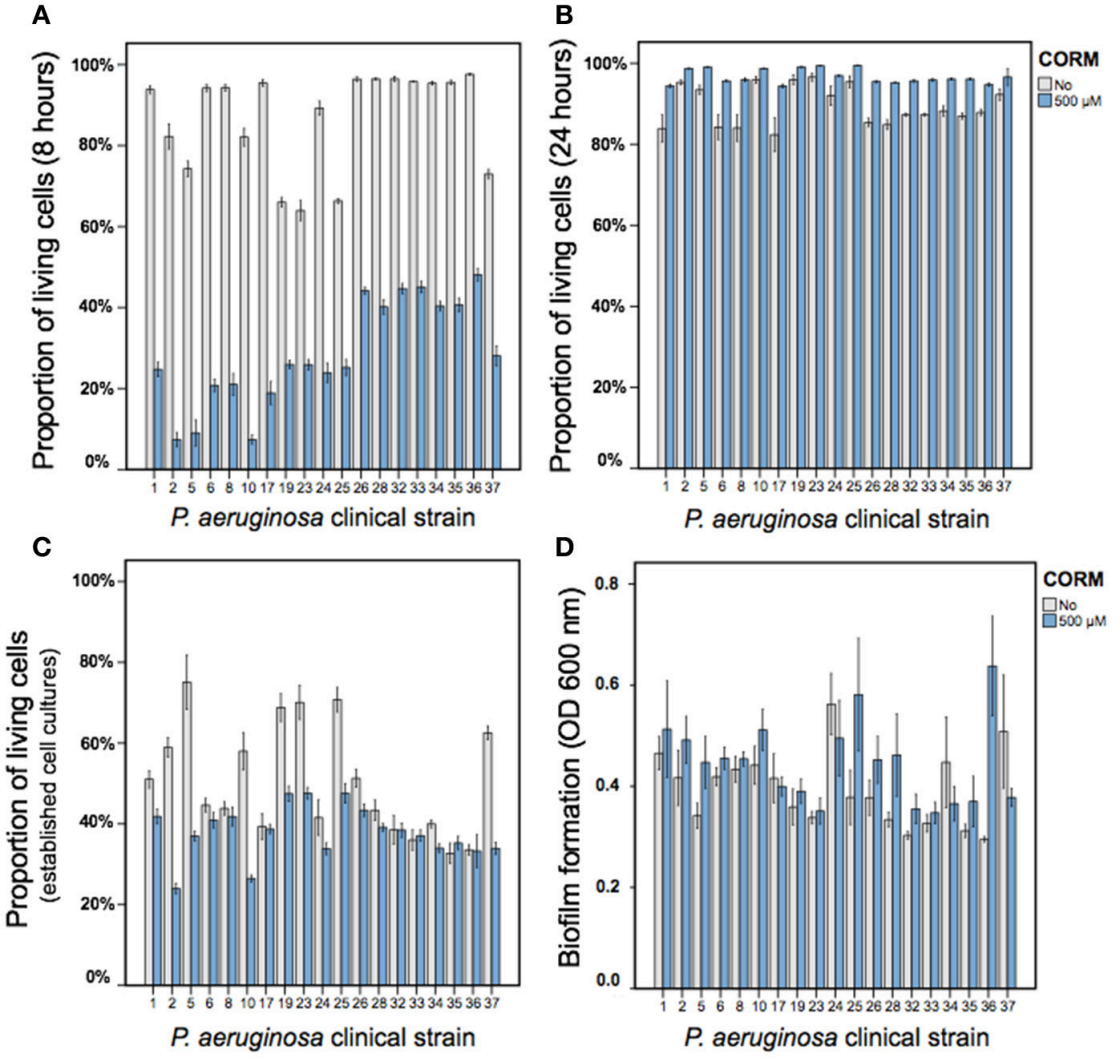
The EBOR-CORM-1 effects on planktonic and biofilm cells of clinical *P. aeruginosa* CF isolates. **(A,B)** show the proportion of living cells after 8 and 24 h of EBOR-CORM-1 application, respectively. **(C,D)** show the EBOR-CORM-1 effects on established cell cultures and biofilms, respectively. In all panels, bars denote for ±1 s.e.m.

Similar to strain PAO1, we found that EBOR-CORM-1 had inhibitory effects on all tested clinical *P. aeruginosa* isolates after 8 h of application of CORM [*F*_(1, 152)_ = 11,969, *p* < 0.001, Figure 3A]. While this effect did not depend on the lineage [CORM × lineage: *F*_(1, 152)_ = 1.4, *p* < 0.001], it varied between different clinical isolates [CORM × isolate: *F*_(18, 152)_ = 11,969, *p* < 0.001, Figure 3A]. In contrast, EBOR-CORM-1 had slightly positive effects on *P. aeruginosa* growth after 24 h of application [*F*_(1, 152)_ = 256, *p* < 0.001, Figure 3B] and this effect varied between different isolates [CORM × strain: *F*_(18, 152)_ = 2.8, *p* = 0.001] being slightly stronger (i.e., positive) with isolates belonging to a lineage B [CORM × lineage: *F*_(1, 152)_ = 24.9, *p* < 0.001, Figure 3B]. EBOR-CORM-1 also had negative effects when applied to established *P. aeruginosa* cell cultures [*F*_(1, 152)_ = 222, *p* < 0.001, Figure 3C]. However, these effects depended on the isolate [CORM × isolate: *F*_(18, 152)_ = 2.8, *p* = 0.001] and the lineage [*F*_(1, 152)_ = 65.2, *p* = 0.001], reduction being relatively larger with isolates belonging to lineage A (Figure 3C). In the case of established biofilms, EBOR-CORM-1 had a slightly positive effect [*F*_(1, 152)_ = 9.6, *p* = 0.002, Figure 3D] and while this effect varied between different isolates [*F*_(18, 152)_ = 2.0, *p* = 0.01] it did not differ between the lineages [*F*_(1, 152)_ = 1.2, *p* = 0.265, respectively, Figure 3D]. Together these results suggest that compared to strain PAO1, EBOR-CORM-1 effects varied more with the clinical *P. aeruginosa* isolates having negative, neutral or positive effects on bacterial growth depending on the isolate identity, lineage and the timing of CORM application.

### Linking EBOR-CORM-1 antimicrobial activity with clinical *P. aeruginosa* isolate virulence and growth

**Figure 4:**
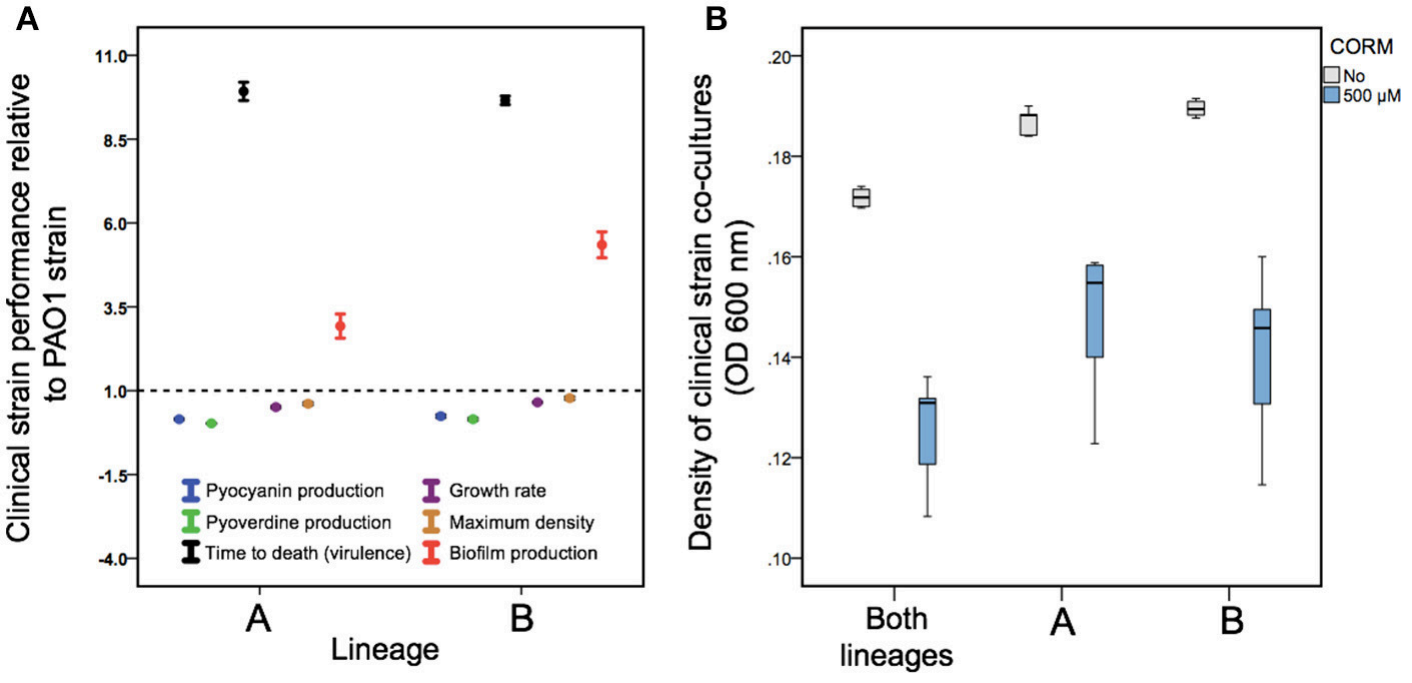
Differences in *P. aeruginosa* growth and virulence trait variation between PAO1 and clinical CF isolates **(A)** and EBOR-CORM-1 effects on clinical CF isolate lineage mixes **(B)**. In **(A)**, different colors denote for pyocyanin (blue) and pyoverdine (green) production, time to death (black), growth rate (purple), maximum density (yellow) and biofilm production for clinical isolates belonging to lineages **(A,B)**. The dashed line shows the mean performance of PAO1 strain. **(B)** shows EBOR-CORM-1 effect on clinical CF isolate mixes after 24 h of CORM application. In **(A)**, bars denote for ±1 s.e.m., and in **(B)**, extreme values around lower and upper quartile (black lines show the median).

We found that all the isolates belonging to a lineage A formed non-mucoid colonies (6 out of 6), while most of the isolates belonging to a lineage B formed mucoid (i.e., mucus-like) colonies (11 out of 13) on LB plates (typical mucoid and non-mucoid colonies shown in Supplementary Figure 3). All clinical isolates differed from the non-mucoid PAO1 strain respective of their virulence and growth (Figure 4A). More specifically, clinical isolates produced less pyoverdine [*F*_(1, 23)_ = 286, *p* < 0.001] and pyocyanin [*F*_(1, 23)_ = 170, *p* < 0.001] and grew slower [*F*_(1, 23)_ = 91, *p* < 0.00] and reached lower maximum densities in LB medium [*F*_(1, 23)_ = 15.5, *p* = 0.001, Figure 4A]. However, clinical isolates produced a considerably larger amount of biofilm [*F*_(1, 23)_ = 21.7, *p* < 0.001] and showed very low virulence (high time to death) in wax moth larvae *in vivo* [*F*_(1, 23)_ = 1296, *p* < 0.001, Figure 4A].

When comparing the two CF lineages, we found that isolates belonging to a lineage B consistently outperformed the isolates belonging to a lineage A by producing more pyoverdine [*F*_(1, 18)_ = 6.06, *p* = 0.025], biofilm [*F*_(1, 18)_ = 15.08, *p* = 0.001] and by growing faster [*F*_(1, 18)_ = 22.35, *p* < 0.001] and to higher maximum densities [*F*_(1, 18)_ = 6.27, *p* = 0.023] in LB medium (Figure 4A; Supplementary Figure 4). However, lineages did not differ in pyocyanin production [*F*_(1, 18)_ = 1.99, *p* = 0.176] or virulence [*F*_(1, 18)_ = 1.03, *p* = 0.324; Figure 4A; Supplementary Figure 4]. Across all clinical isolates, density reduction by CORM correlated negatively with biofilm formation [*F*_(1, 18)_ = 4.8, *p* = 0.042]. Together these results suggest that clinical isolates differed from PAO1 and from each other respective to various life-history traits important for establishing an infection.

### EBOR-CORM-1 activity against clinical *P. aeruginosa* CF isolate co-cultures

Despite the observed isolate-specific variation in *P. aeruginosa* monocultures, EBOR-CORM-1 was effective in reducing the growth of *P. aeruginosa* co-cultures after 24 h of application [CORM: *F*_(1, 24)_ = 132, *p* < 0.001, Figure 4B]. Moreover, this reduction was the same regardless of whether the mix contained only one lineage or both lineages [CORM × co-culture: *F*_(2, 24)_ = 0.5, *p* = 0.612]. These results suggest that intraspecific *P. aeruginosa* population heterogeneity makes the bacteria more susceptible to EBOR-CORM-1 treatment.

### EBOR-CORM-1 activity against *P. aeruginosa* strains in wax moth model

**Figure 5:**
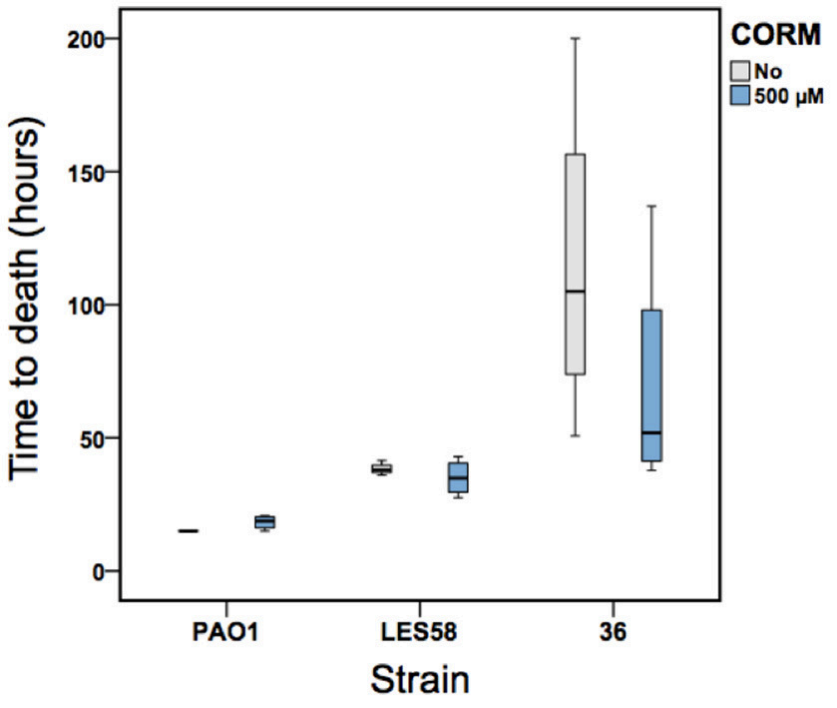
The EBOR-CORM-1 activity against three *P. aeruginosa* strains in wax moth model. Boxplots show larval survival in the absence (light gray) and presence (blue) of EBOR-CORM-1 for PAO1, LESB58 and clinical isolate #36 (lineage B). Bars show extreme values around lower and upper quartile and black lines show the median.

We found that *P. aeruginosa* isolates differed in their virulence (time to death) from each other [*F*_(2, 24)_ = 12.2, *p* < 0.001]: PAO1 and LESB58 strains were equally virulent, and both exhibited higher virulence than the clinical isolate 36 (killing larvae approximately in 17 h [PAO1], 36 h [LESB58], and 92 h [clinical isolate 36]; values averaged over both non-CORM and CORM treatments, Figure 5). In contrast to *in vitro* results, application of EBOR-CORM-1 did not increase the survival of infected larvae [*F*_(1, 24)_ = 1.3, *p* = 0.257] with any of the infected strains [CORM × strain: *F*_(2, 24)_ = 1.4, *p* = 0.273, Figure 5]. All larvae became highly pigmented (black throughout) during the infection regardless of the *P. aeruginosa* isolate.

## Discussion

Here we set out to study the antimicrobial activity of [NEt_4_][MnBr_2_(CO)_4_], EBOR-CORM-1, against clinical *P. aeruginosa* isolates *in vitro*. This CORM was chosen as a suitable representative of this class of molecule based on the aqueous solubility, facile synthesis (Angelici, 1964), content of a non-toxic metal core, and simple architecture which makes it akin to a “parent compound” for CORMs that have been engineered to possess sophisticated CO release mechanisms. In contrast to more complex CORMs, the molecule was shown to have a water activated mechanism of CO release, as seen in previous studies of [MX(CO)_5_]^−^ species, where X is a halide (Zhang et al., 2009). Such water induced degradations are believed to proceed via a two-step pathway whereby water causes loss of the halide followed by formation of a dimer species; from which the CO is released. This may explain the changes in the IR spectra recorded in water when compared to chloroform, although the data do not directly match those for [Mn_2_Br_2_(CO)_8_] (El-Sayed and Kaesz, 1963), the product expected on loss of Br^−^ from EBOR-CORM-1. We found that while EBOR-CORM-1 showed density-dependent antimicrobial activity against both planktonic and biofilm cells of the widely studied laboratory-adapted strain PAO1, these effects were more varied and weaker against clinical CF lung isolates. Regardless, EBOR-CORM-1 was efficient at reducing the growth of CF isolate lineage mixes, which suggests that it could have therapeutic potential in controlling heterogeneous *P. aeruginosa* infections. Solutions of inactivated EBOR-CORM-1 were essentially inactive against *P. aeruginosa* strain PAO1 (Supplementary Figure 2) implying that, at least in this case, the observed activity was due to CO released from the complex rather than the residual metal salts (or indeed [NEt_4_]^+^).

Similar to a study published by Murray et al. (2012), we found considerable variation in CORM antimicrobial activity between different clinical CF isolates, which depended whether we explored EBOR-CORM-1 effects on relatively short (8 h) or long timescales (24 h) and if we compared CORM antimicrobial activity on actively growing and established cell cultures (after 48 h of bacterial growth). Our results after 8 h of EBOR-CORM-1 application are very similar to a previous study (Murray et al., 2012) showing clear reduction in bacterial densities. However, this effect vanished by the 24 h time point, and surprisingly, some bacterial isolate cultures reached higher optical densities in the presence compared to absence of CORM, which could have been due to increase in number of cells or expression of exoproducts that were picked up by OD600 nm (e.g., pyocyanin or alginate). The most likely explanation for this is that CORM effects were short-lived (Figure 1), which allowed bacteria to recover and grow to high densities during 24 h after application of CORM. However, when CORM effects were measured after 4 h of application to established cell cultures, we could still observe clear reduction in mean bacterial densities. Together these results suggest that CORM effects could be seen up to 4 h post application and that CORM could eradicate bacterial cells whether they are at exponential or stationary phase of their growth. Interestingly, CORM effects varied between clinical isolates and were clearer with the isolates belonging to lineage A. While Murray et al. (2012) did not observe clear variation in CORM effects against planktonic cell cultures, they found differences in CORM efficiency in eradicating bacterial biofilms. This is also consistent with our data and reinforces the hypothesis that *P. aeruginosa* clinical isolates are likely to respond differently to CORM therapies.

To explore clinical isolate variation in more detail, we compared differences in bacterial virulence and growth traits between the PAO1 and clinical CF lung isolates. We found that relative to strain PAO1, clinical CF isolates grew slower, had lowered virulence and produced lower amounts of pyoverdine and pyocyanin, which are important virulence factors (O'Brien et al., 2017). This is consistent with previous research and typical for *P. aeruginosa* isolates retrieved from chronic lung infections (Smith et al., 2006; Folkesson et al., 2012; Marvig et al., 2013; Williams et al., 2015). The clinical isolates produced much more biofilm compared to strain PAO1 and biofilm formation was the highest in the isolates belonging to lineage B. Biofilms could potentially provide a protective function against CORMs. Biofilms often have much higher antibiotic resistance than their aquatic counterparts (Stewart and William Costerton, 2001) and there are multiple reasons for this. First, antibiotics might be ineffective because the biofilm acts as a diffusion barrier (de Beer et al., 1997). Second, subpopulations within the biofilm can sometimes differentiate into a highly protected phenotypes that can repopulate the biofilms (Cochran et al., 2000). Third, the biofilm might change the chemical microenvironment, forming zones of nutrient and oxygen depletion or waste accumulation that prevents the antibiotics from functioning optimally (de Beer et al., 1994). Although we did not explore this specifically, clinical isolates belonging to a lineage A were more susceptible to CORMs and produced relatively less biofilm compared to strains belonging to a lineage B. Thus, overall a negative correlation was found between density reduction by CORM and biofilm formation. Our results therefore suggest that biofilm might provide a protective function against the CORM.

Despite the isolate variations observed in bacterial monocultures, EBOR-CORM-1 was effective at reducing the growth of *P. aeruginosa* clinical isolate mixed cultures. One explanation for this is that, in addition to CORM, *P. aeruginosa* growth was limited by antagonistic intraspecific species interactions in co-cultures. *P. aeruginosa* has been shown to exert both facilitative and antagonistic effects on each other via siderophore (Harrison et al., 2008) and bacteriocin (Ghoul et al., 2015) production. In our case, all the clinical isolates were derived from the same Liverpool Epidemic Strain clonal lineage and therefore likely carried the same siderophore and bacteriocin genes. Additionally, resource competition is likely to further limit *P. aeruginosa* growth both in CF lungs and simplified laboratory microcosms. As a result, even though some clinical strains were relatively insensitive to EBOR-CORM-1, their growth could have been constrained by competition with the other strains in co-cultures. We found that this was the case for all strain mixes regardless if the strains belonged to a lineage A, B or them both. This suggests that the susceptibility of the lineages measured in monocultures did not predict the susceptibility of isolate mixes within or between lineages. However, such antagonism was not observed in the absence of EBOR-CORM-1, which suggests that CORM-triggered antagonistic intraspecific interactions in *P. aeruginosa* co-cultures. Mechanistically, this could have been driven by competition sensing in response to CORM-mediated cell damage in *P. aeruginosa* populations (Cornforth and Foster, 2013). However, this needs to be confirmed in future experiments. Interestingly, all the clinical strains we used originated from a single CF patient and interactions between them thus reflect the realistic ecology of CF lungs. In the future, it would be useful to determine pairwise interactions between these CF strains and look at CORM effects on other coexisting bacterial species observed in CF infections (Folkesson et al., 2012).

We found that EBOR-CORM-1 had no clear therapeutic benefits in the wax moth infection model. There are several potential explanations for this. First, EBOR-CORM-1 had limited long-term activity when in contact with water. As a result, the bactericidal effect may only have elicited lag in the initial phase of bacterial growth and proliferation within the wax moths. Second, insect tissue is not homogeneous and it is possible that we failed to deliver the CORM to the specific area of infection, or that bacteria were able to colonize new areas that were not exposed to the CORM. Third, insects differ from laboratory media (such as LB) as a bacterial growth environment, which could also affect pathogen virulence. For example, it has been recently demonstrated that plant vs. animal based growth media can have physiological effects on bacterial virulence (Ketola et al., 2016) and that LB media does not adequately reflect *P. aeruginosa* growth on lung tissue (Harrison et al., 2014; Harrison and Diggle, 2016). Hence, the wax moth injection model might not reliably reflect the virulence of CF isolates derived from chronic infections. However, it is also the case that many of the affordable and available CF infection animal models do not truly reflect the real CF lung disease environment. It remains to be established whether CORM therapy could be applied in the context of CF lung infections. It is possible, for example, that it might be more suitable for treating topical infections such as burn wounds, for which better animal models are available (Rumbaugh et al., 2012).

Further work is also needed to understand the mode of action of EBOR-CORM-1. While respiratory oxidases and globins at heme targets are generally considered the prime targets of CO and CORMs (Wareham et al., 2015), it has been demonstrated that CORMs can have multiple different other targets (Wilson et al., 2015). For example, CO also binds to the di-iron site in bacterial NO reductases and to iron, copper, and nickel sites in certain microbial proteins such as CO dehydrogenase (Lu et al., 2004; Wasser et al., 2005). In some cases, CORMs might have intracellular targets but their accumulation within the cells can be very weak (Tinajero-Trejo et al., 2016). Moreover, in the future it would be important to test if EBOR-CORM-1 is cytotoxic to eukaryotic cells. The concentration we used are in line with previously published work where no, or very mild, cytotoxic effects were observed (Murray et al., 2012). We are currently conducting experiments to validate this independently and to understand how EBOR-CORM-1 interacts with bacterial cells. While, our wax moth assays show that the concentrations we used had no negative effects on short-term insect viability, more detailed cytotoxicity assays are needed in the future. Lastly, the low solubility of EBOR-CORM-1 in water, and its activation in this medium, is problematic for delivery and activation at specific sites within patients. In addition to chemically increasing the molecule stability, CORMs could be enclosed in microvesicles (van Dommelen et al., 2012) to ensure more efficient antimicrobial activity and drug delivery.

In conclusion, our results show that EBOR-CORM-1 shows antimicrobial activity against both planktonic and biofilm cells of *P. aeruginosa* strain PAO1 but that these effects are more varied and less pronounced against clinical CF lung isolates in monocultures. In contrast, more heterogeneous *P. aeruginosa* populations comprising intraspecific phenotypic variants were more susceptible to CORM treatment. This potentially has wider implications in the testing of novel therapeutics. At present, this is done almost exclusively using clonal *P. aeruginosa* populations. Our observations suggest that testing carried out on more heterogeneous populations of *P. aeruginosa*, more closely resembling those found in the CF lung, may give different and sometimes more promising results.

## Author contributions

LF, RS, KS, MK, BA, CW, IF, JL, AP, and V-PF designed and performed the experiments. LF and V-PF analyzed the data and all authors wrote the manuscript.

### Conflict of interest statement

The authors declare that the research was conducted in the absence of any commercial or financial relationships that could be construed as a potential conflict of interest.
