# Supplementary material for: The Antimicrobial Activity of a Carbon Monoxide Releasing Molecule (EBOR-CORM-1) Is Shaped by Intraspecific Variation within Pseudomonas aeruginosa Populations
Source: Front Microbiol. 2018 Feb 8;9:195. doi: 10.3389/fmicb.2018.00195 (PMC5809400; doi:10.3389/fmicb.2018.00195)
Supplement: Supplementary file 1 [file DataSheet1.docx]

**Supplementary material**

**
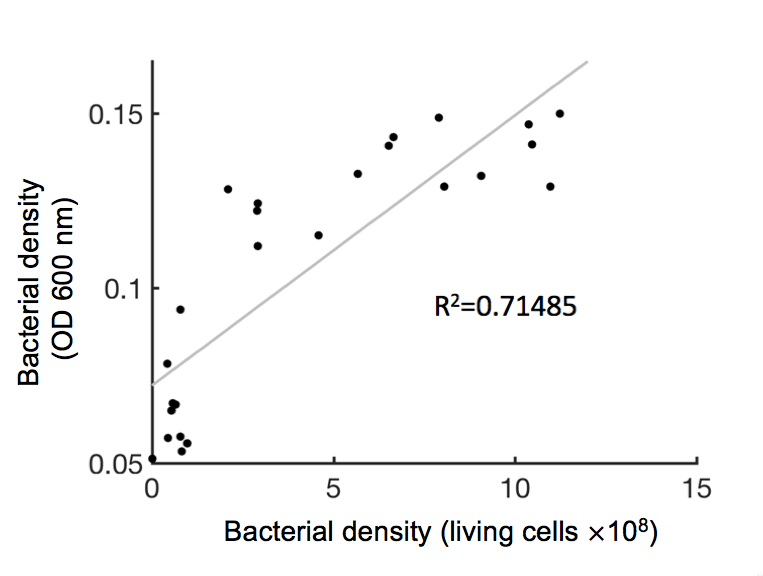
**

**Supplementary figure 1.** Correlation between optical density (OD 600 nm) and number of living cells (flow cytometry) for *P. aeruginosa* PAO1 strain.


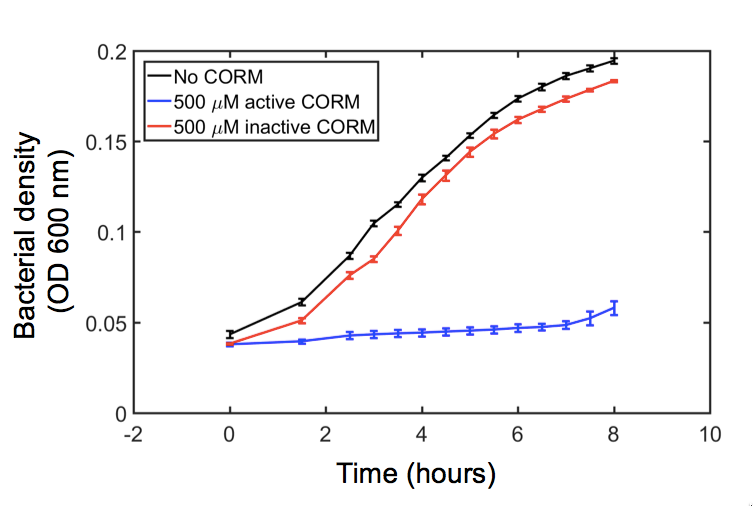


**Supplementary figure 2.** Comparison of active and inactivated (after 24 h solubilisation into dH_2_O at room temperature at 22 °C) EBOR-CORM-1 against *P. aeruginosa* PAO1 strain. Bars show ± standard error of mean.

**
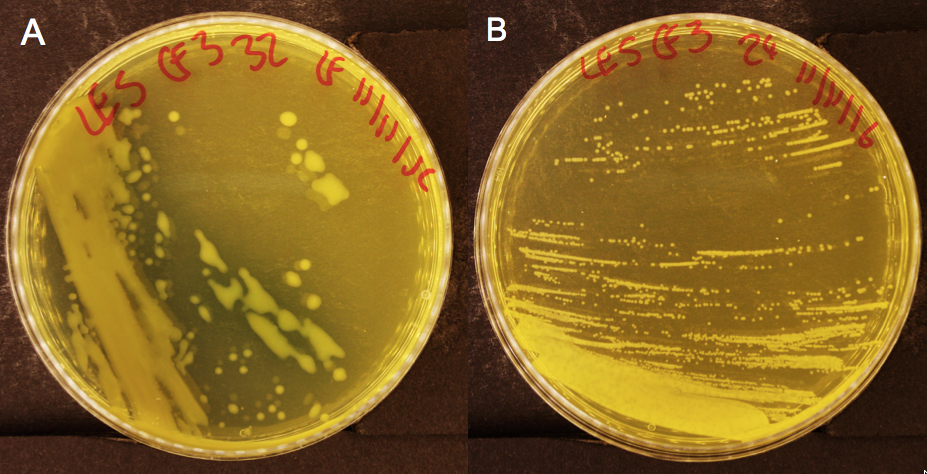
**

**Supplementary figure 3.** Example photos of mucoid (panel A) and non-mucoid (panel B) *P. aeruginosa* CF strains.


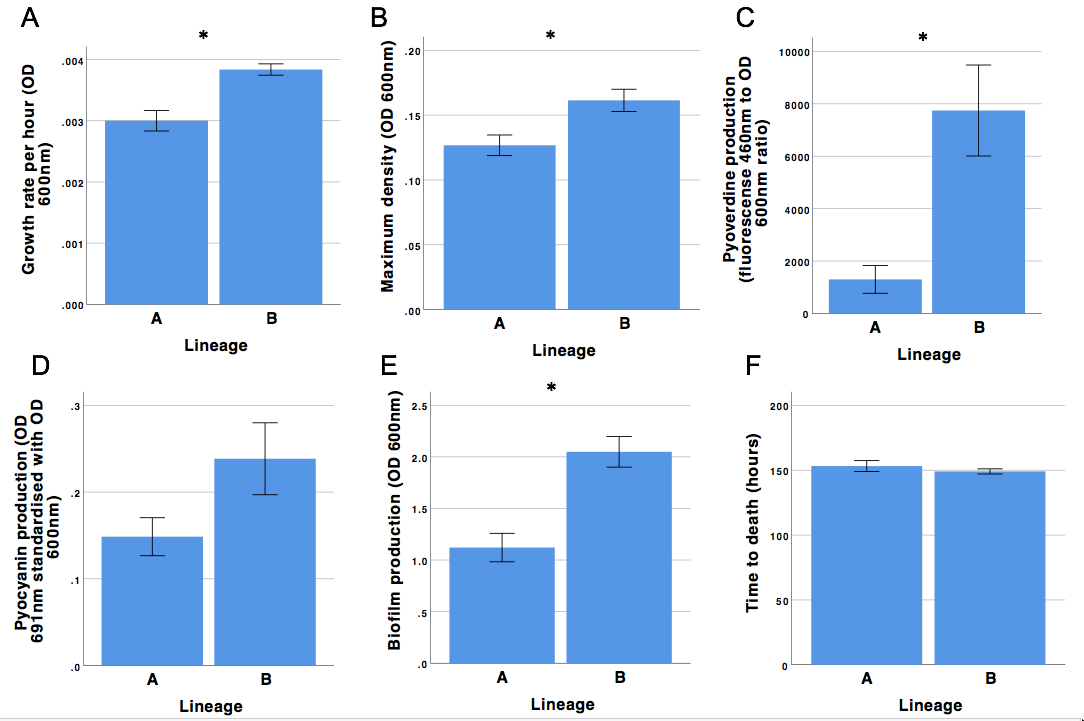


**Supplementary figure 4.** Differences in the virulence of clinical CF isolates belonging to lineages A and B. Panels A to F show differences in growth rate, maximum density, pyoverdine production, pyocyanin production, biofilm production and virulence measured *in vivo* in wax moths (time to death), respectively. In all panels, bars denote for ±1 s.e.m. and stars denote for statistically significant differences between lineages A and B.
